# Long COVID symptoms and demographic associations: A retrospective case series study using healthcare application data

**DOI:** 10.1177/20542704241274292

**Published:** 2024-08-28

**Authors:** David Sunkersing, Henry Goodfellow, Yi Mu, Mel Ramasawmy, Mayur Murali, Lawrence Adams, Ted J FitzGerald, Ann Blandford, Fiona Stevenson, Julia Bindman, Chris Robson, Amitava Banerjee

**Affiliations:** 1 4919 University College London, London, UK; 2 Living With Ltd, London, UK

**Keywords:** Patients, non-clinical, population trends, epidemiology, non-clinical, telemedicine, health informatics, non-clinical

## Abstract

**Objectives:**

To investigate long COVID (LC) symptoms self-reported via a digital application. Explore associations between various demographic factors and intensity of LC symptoms.

**Design:**

A retrospective case series study. We analysed self-reported symptoms from 1008 individuals with LC between November 30, 2020, and March 23, 2022.

**Setting:**

England and Wales.

**Participants:**

Individuals with LC using the healthcare application in 31 post-COVID-19 clinics and self-reporting LC symptoms.

**Main outcome measures:**

Highest reported LC symptoms, associations with demographic factors and intensity of symptoms.

**Results:**

109 symptom categories were identified, with pain (26.5%), neuropsychological issues (18.4%), fatigue (14.3%) and dyspnoea (7.4%) the most prevalent. The intensity of reported symptoms increased by 3.3% per month since registration. Age groups 68–77 and 78–87 experienced higher symptom intensity (32.8% and 86% higher, respectively) compared to the 18–27 age group. Women reported 9.2% more intense symptoms than men, and non-white individuals with LC reported 23.5% more intense symptoms than white individuals with LC. Higher education levels (national vocational qualification (NVQ) 3 to NVQ 5) were associated with less symptom intensity (27.7%, 62.8% and 44.7% less, respectively) compared to the least educated (NVQ 1–2). People in less deprived areas had less intense symptoms than those in the most deprived area. No significant association was found between index of multiple deprivation (IMD) decile and number of symptoms.

**Conclusion:**

Treatment plans must prioritise addressing prevalent LC symptoms; we recommend sustained support for LC clinics. Demographic factors significantly influence symptom severity, underlining the need for targeted interventions. These findings can inform healthcare policies to better manage LC.

Long COVID (LC), also known as Post-Acute Sequelae of SARS-CoV-2 Infection, is an illness in which individuals with LC continue to experience symptoms months after recovering from COVID-19.^1^ Symptoms of LC differ widely and can include fatigue, brain fog, shortness of breath and chest pain.^2^

The prevalence of LC varies depending on the study design and population studied. However, the current prevalence is estimated to be around 10–30% of non-hospitalised people who have had COVID-19, 50–70% of hospitalised cases and 10–12% of vaccinated cases.^3^

The long-term effects of COVID-19 are still being studied, and the incidence rate of LC may change over time. In the UK, studies have explored LC symptoms and risk factors in non-hospitalised individuals using primary care records^4^ and consolidated evidence on persistent symptoms and their associations in broader populations.^5^ Additionally, there has been significant interest in Patient-Reported Outcome Measures. One study measured LC symptoms important to patients, aiming to aid in intervention evaluation and clinical management.^6^ Another study aims to assess LC symptom burden, underlying pathophysiology and evaluate potential therapies in non-hospitalised individuals.^7^ Importantly, current evidence suggests that LC is relatively common, highlighting the need for healthcare providers to be aware of it and support individuals with LC.

In response to the COVID pandemic and the emergence of LC, a digital health intervention (DHI) featuring a clinician-prescribed, patient-facing application (app) (Living With COVID Recovery [LWCR]) was developed and deployed,^8^ enabling individuals with LC to follow prescribed treatments, set goals, understand their condition and measure their progress. Amongst other features, users could self-report their symptoms using free-text into the app (i.e. not selecting from a list of pre-defined or suggested symptoms) and assign an intensity level between 0 (which app users were told represented ‘not at all intense’) and 10 (which app users were told represented ‘extremely intense’) of each self-reported symptom.

Although several mobile health apps were designed and deployed during the COVID-19 pandemic, the majority were designed to track acute COVID-19 symptoms, with very few addressing long-term symptoms.^9^ Moreover, despite a recent increase in LC research, the aetiology, clinical characteristics and optimal management of LC remain complex and elusive. Furthermore, the risk factors and predictors of LC remain poorly defined, making it challenging to identify individuals at the highest risk and implement preventive measures. In particular, there is currently limited research on the association between various demographic factors and intensity of LC symptoms.

As such, this study aims to examine individuals with LC's self-reported symptoms of LC, identifying their prevalence in this sample and relationship between demographic factors (age, gender, ethnicity and indices of multiple deprivation [IMD] decile) and the intensity of LC symptoms. We also highlight that the novelty of this work lies in its early-pandemic, nationwide self-reported data collection. Our study complements and adds to existing research into greater understanding LC – and could inform the development of effective management strategies and targeted interventions aimed at addressing LC.

## Methodology

### Design and setting

Individuals with LC using the LWCR DHI as part of their assessment and treatment in 31 specialised post-COVID-19 clinics in England and Wales.

### Intervention

The LWCR DHI has been purposefully designed to be part of clinical treatment in post-COVID-19 clinics. Further information on this DHI can be found in previously published literature.^10,11^

### Study participants

Individuals with LC using the LWCR DHI.

These individuals with LC had been referred to an NHS post-COVID-19 clinic in England and Wales from primary or secondary care settings, having experienced LC symptoms lasting ≥12 weeks. Individuals with LC deemed eligible for the LWCR DHI had to meet the following criteria: aged 18 years or older, had access to a smartphone, were expected to benefit from the intervention, were physically capable of rehabilitation and had proficiency in the English language.

All eligible individuals with LC had registered on the LWCR DHI between November 30, 2020, and March 23, 2022 (*n* = 3833). All data collected in the LWCR product were pseudo-anonymised using a unique patient ID number and were stored in Metabase (www.metabase.com).

### Statistical analysis

A total of 1604 (unique) self-reported symptoms from 1008 individuals with LC forming 3340 patient-symptom combinations using the LWCR app (considering only the first (baseline) measure of each self-reported symptom per patient) were included in this study.

All self-reported symptoms were clinically interpreted by five clinicians until agreement in their clinical classification was met. For example, ‘Anxiety’, ‘Anxiety and Fear’, ‘Crying’, ‘Depression’ and ‘Fear’ were all classified as ‘Neuropsychological’ symptoms.

Following this, using (Stata/MP 17.0), the analysis was conducted using a multiple linear regression model that regressed intensity of symptom on the following variables:

- Months since registration represents the time gap (in months) between the first time a patient reported a specific symptom and the time when the patient registered for the app. (mean 0.95, standard deviation 1.66, min 0, max 12.32).
- Age categories (18–27, 28–37, 38–47, 48–57, 58–67, 68–77, 78–87).
- Gender (male and female).
- Ethnicity (white and non-white).
- Education (NVQ1-2, NVQ 3, NVQ 4 and NVQ 5) where NVQ 1-2 represents the least educated level (a school leaver), NVQ 3 represents A level, NVQ 4 represents Degree level and NVQ 5 represents Postgraduate Degree level. (NVQ = national vocational qualification).
- IMD decile (1–10) where 1 represents the most deprived areas.

Descriptive statistics are shown in Table 1.

**Table 1.** Descriptive statistics.

	Number of individuals with LC	Percentage of total individuals with LC (%)	Number of unique symptoms	As a % of total unique symptoms
**Number**	1008		1604	
**Age**				
18–27	50	5.0	104	6.5
28–37	151	15.0	305	19.0
38–47	284	28.2	571	35.6
48–57	356	35.3	627	39.1
58–67	142	14.1	297	18.5
68–77	20	2.0	34	2.1
78–87	5	0.5	9	0.6
**Sex**				
Male	250	24.8	458	28.6
Female	753	74.7	1209	75.4
Unspecified	5	0.5	38	2.4
**Ethnicity**				
White	817	81.1	1342	83.7
Non white	80	7.9	177	11.0
Unspecified	111	11.0	220	13.7
**Education**				
NVQ 1–2	168	16.7	329	20.5
NVQ 3	169	16.8	365	22.8
NVQ 4	239	23.7	449	28.0
NVQ 5	308	30.6	551	34.4
Unspecified	124	12.3	228	14.2
**IMD Decile**				
1	15	1.5	33	2.1
2	71	7.0	252	15.7
3	63	6.3	131	8.2
4	96	9.5	219	13.7
5	106	10.5	175	10.9
6	106	10.5	228	14.2
7	120	11.9	234	14.6
8	97	9.6	242	15.1
9	109	10.8	204	12.7
10	111	11.0	195	12.2
Unspecified	114	11.3	227	14.2

Intensity of the symptoms was originally a 0–10 scale, where ‘0’ represented very low intensity and ‘10’ represented very high intensity. For the ease of interpretation, we have standardised this variable to have a mean of 0 and a standard deviation (S.D.) of 1 (min: −2.66; max: 1.46).

## Results

Of the 1008 individuals with LC, 233 (23%) reported symptoms only once (i.e. individuals with LC reported only one symptom and that symptom was reported only once), while 775 (77%) reported symptoms multiple times. The most prevalent symptoms reported were pain (26.5% of all symptoms reported; reported by 44.4% of individuals), neuropsychological (18.4% of all symptoms reported; by 40.7% of individuals), fatigue (14.3% of all symptoms reported; reported by 42.5% of individuals) and dyspnoea (7.4% of all symptoms reported; reported by 21.3% of individuals). Other prevalent symptoms included palpitations, light-headedness and tinnitus. A breakdown of pain symptoms used is in Appendix 1. A breakdown of neuropsychological symptoms is in Appendix 2.

**Figure 1.**
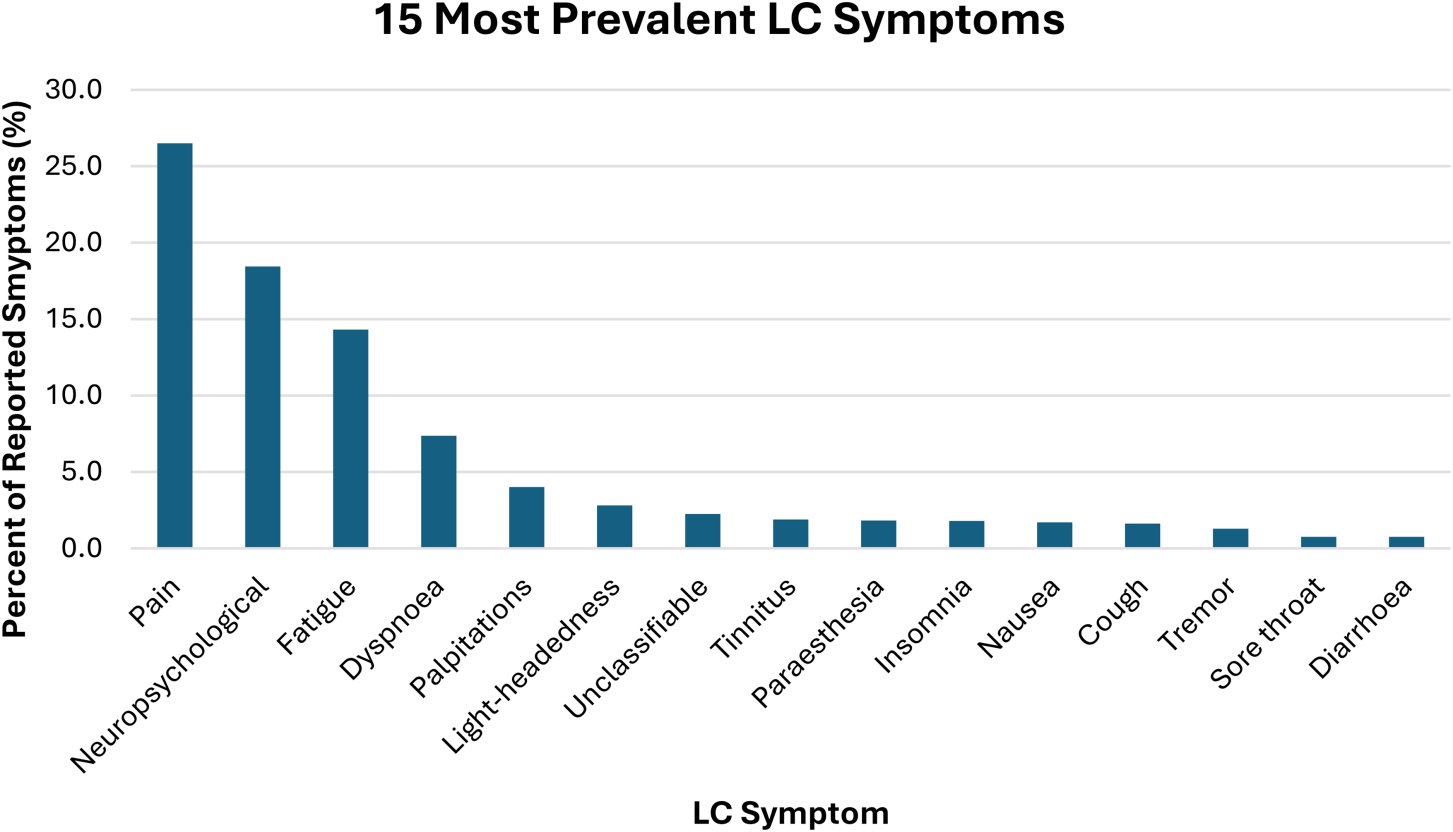
Fifteen most prevalent LC symptoms.

After clinical classification, 109 symptom categories were identified (Appendix 3). The 15 most prevalent symptoms (where unique self-reported symptoms are considered) are displayed in Figure 1:

The multiple linear regression model showed:

- The intensity of reported symptoms is positively associated with time between a patient's registration to the app and the initial reporting of symptoms (keeping all the other factors the same, i.e. age, gender, ethnicity, IMD and education).
- Age, gender, ethnicity, education and IMD decile were significantly associated with intensity of symptoms (keeping other variables constant).
- No statistically significant association was found between IMD decile and number of symptoms.

A summary of these results is shown in Table 2; a detailed written summary in Appendix 4:

**Table 2.** Summary of factors associated with symptom intensity from multiple linear regression analysis.

Factor	Comparison group	% Change in symptom intensity (s.d.)	*p* value	Change in symptom intensity (0–10 scale)	*p* value
Time since registration (per 1 month)	-	+3.3%	<0.01	+0.1	<0.01
Age 68–77 vs. Age 18–27	Age 18–27	+32.8%	<0.1	+0.8	<0.1
Age 78–87 vs. Age 18–27	Age 18–27	+86%	<0.05	+2.1	<0.05
Gender (Women vs. Men)	Men	+9.2%	<0.05	+0.2	<0.05
Ethnicity (Non-white vs. White)	White	+23.5%	<0.01	+0.6	<0.01
Education NVQ 3 vs. NVQ 1–2	NVQ 1–2	−27.7%	<0.01	−0.7	<0.01
Education NVQ 4 vs. NVQ 1–2	NVQ 1–2	−62.8%	<0.01	−1.5	<0.01
Education NVQ 5 vs. NVQ 1–2	NVQ 1–2	−44.7%	<0.01	−1.1	<0.01
IMD decile 3 vs. IMD decile 1	IMD decile 1	−46.6%	<0.01	−1.1	<0.01
IMD decile 5 vs. IMD decile 1	IMD decile 1	−52.7%	<0.01	−1.3	<0.01
IMD decile 10 vs. IMD decile 1	IMD decile 1	−68.6%	<0.01	−1.7	<0.01

## Discussion

In this app-based, retrospective study across three waves of the pandemic, we found that pain, neuropsychological symptoms and fatigue were the commonest self-reported symptoms in individuals with LC in England and Wales.

Our finding that pain was the most reported is consistent with the literature.^3,12^ From abdominal pain to eye pain and neuropathic pain, the spectrum of pain manifestations in LC is diverse, underscoring the complexity of this condition. Our study highlights that when pain was considered as a broad category of symptom (see Appendix 1 for categories), this was the most self-reported symptom of LC at 19.6%. Acknowledging the subjectivity inherent in clinical symptom classification, we recognise that our list may not be exhaustive and may require expansion. The nuanced nature of pain perception by individuals with LC in the app underscores the complexity of symptomatology, emphasising the need for ongoing refinement and expansion of our understanding to better capture the diverse ways in which their health challenges are articulated and experienced. Nevertheless, our findings reinforce the importance of recognising and addressing pain as a crucial component of the potential long-term consequences of COVID-19.

Our finding that neuropsychological symptoms (e.g. anxiety and/or depression) are top self-reported symptoms of LC is consistent with current literature on COVID-19's mental health effects. Individuals who have had COVID-19 are at increased risk of developing anxiety and other mental health problems, including depression and post-traumatic stress disorder.^13^ A systematic review found a 27.77% prevalence of anxiety disorders in people with LC.^14,15^ We would consider this notable, given the 1–7% lifetime prevalence of anxiety disorders in the general population of Europe.^16^ Clinicians should screen for anxiety in all individuals with LC and collaborate on tailored treatment plans, including medication, therapy and lifestyle changes. This finding highlights the importance of developing effective anxiety management strategies and suggests that stress reduction and coping interventions may benefit individuals with LC.

Fatigue, characterised by an overwhelming sense of tiredness and weakness, stands out in our study as a predominant and persistent symptom among individuals with LC. This is consistent with previous studies reporting this as one of the commonest LC symptoms.^3^ Even months after the initial infection, many individuals report experiencing profound exhaustion, often disproportionate to the level of physical or mental exertion. This chronic fatigue not only hampers the ability to engage in routine activities but also poses significant hurdles to the overall well-being and recovery of those affected.^17^ This finding highlights the need for ongoing research and comprehensive care strategies to address this ongoing challenge faced by individuals with LC.^3^

We found dyspnoea to be a top self-reported symptom of LC, consistent with current literature on the long-term effects of COVID-19. Research indicates that people who have had COVID-19 face heightened risk of respiratory issues.^18^ Additionally, recent data from the Office for National Statistics reported that 48% of individuals with LC self-reported breathlessness symptoms.^19^

The high prevalence of self-reported symptoms being labelled as ‘unclassifiable’ by our five clinicians signifies a significant challenge in interpreting and categorising the diverse range of reported experiences. Many individuals describe symptoms that fall outside conventional medical classifications, and some entries may reflect not only genuine but also subjective or non-specific expressions of their health status. It is plausible that participants, unfamiliar with the app interface or the clinical nuances of symptom description, may use words that do not readily align with established medical terms.^20^ This highlights the crucial need for a nuanced understanding of how individuals with LC use the app, emphasising the importance of interviews or walkthroughs to contextualise symptoms.^21^ Understanding the perspectives of individuals with LC and ensuring clarity in how they use the app can enhance the accuracy and relevance of self-reported data, leading to a more meaningful comprehension of LC's complex symptomatology.

Our study found a significant positive association between the intensity of reported symptoms and the duration between a patient's registration on the app and their initial symptom report. One interpretation is that individuals with LC become more attuned to, or comfortable in expressing, the severity of their symptoms as they navigate the app (or that worsening symptoms prompt self-reporting). Similar findings in literature have found that disease severity increased the likelihood of a person seeking healthcare.^22^ Understanding the underlying mechanisms driving this association is crucial for refining the app's functionality and tailoring interventions to address evolving patient needs, including psychological and behavioural factors influencing symptom reporting. Further research is warranted to delve deeper into these dynamics and inform strategies for optimising the effectiveness of app-based healthcare interventions.

Our results additionally demonstrated a significant relationship between age and the intensity of LC symptoms. Specifically, a positive relationship was found between age and intensity of LC symptoms. This suggests that older individuals may experience a greater severity of symptoms compared to their younger counterparts, highlighting the need for targeted interventions and support for this population. This finding is consistent with published research that suggests the risk of developing LC increases with age.^23^

Several reasons may explain why older adults may be more at risk for LC. One possibility is that their immune systems are weaker, making them more susceptible to the virus and its long-term effects. Another possibility is that they have more underlying health conditions, which can make them more vulnerable to the effects of COVID-19. Although pre-hospitalisation status was not included at the time of this study within the app, we recognise the potential value of such information in enhancing our analysis depth. The finding that LC symptoms are more intense in older adults is a significant concern. More research is needed to understand underlying LC mechanisms and to develop effective treatments for all age groups.

In contrast to age, no relationship was found between gender and intensity of LC symptoms. Further research is needed to explore potential gender-related factors^24^ influencing LC symptoms.

We also found that ethnicity emerged as a significant factor impacting the intensity of LC symptoms, with ‘non-white’ individuals experiencing more intense symptoms. This finding raises important considerations regarding health disparities and suggests that individuals from non-white ethnic backgrounds may experience more severe LC symptoms. We note that this is consistent with the current findings in literature.^25^ Significantly fewer ‘non-white’ individuals self-reported symptoms than ‘white’ individuals – despite experiencing more intense symptoms. The disparity suggests potential barriers to the utilisation of digital healthcare (and potential digital divide) within certain demographic groups, particularly for those with more intense symptoms. Further research is warranted to understand these disparities and develops targeted interventions for affected communities.

Our study additionally highlights a significant association between education levels and the intensity of symptoms, with higher education levels being linked to less intense symptoms. This finding is consistent with that in the literature, where higher educational levels have been associated with lower symptom intensities.^26^ This observation suggests that education may play a role in influencing the self-reported severity of symptoms, suggesting a potential correlation between educational attainment and mental or physical well-being. We must also consider that education level may also be associated with various socio-economic factors (e.g. income, housing and access to resources), which could have a more direct influence on the intensity of symptoms.^27^

This study found that for this population, higher IMD decile scores were associated with a higher intensity of LC symptoms. This suggests that individuals in more deprived areas may be more susceptible to severe LC symptoms. This finding is consistent with and an extension of current findings, with evidence that people from the most socio-economically deprived populations have the highest risk of LC.^28^ Addressing the social determinants of health and providing equitable access to healthcare resources may help mitigate the impact of LC. No statistically significant association was found between IMD decile and number of symptoms.

We must also consider an individual's perception of intensity – and that this is a complex phenomenon influenced by several factors, including psychological, physiological and environmental variables.^29^ Thus, when considering our findings, we must also consider that there are individual differences in intensity perception. These all play crucial roles in shaping how people experience their symptoms and this should be considered when individuals in this study self-reported their LC symptoms.

One major limitation of this study is that we do not have information about the co-morbidities, pre-hospitalisation or vaccine status of the individuals with LC. This is an important limitation as individuals with certain co-morbidities or who were previously hospitalised or not vaccinated at the time of self-reporting their symptoms may have been at greater risk of developing specific symptoms. Future studies should aim to collect this information to better understand the complex relationship between LC and other health factors.

This study may introduce bias against individuals who lack technological competency or familiarity. Those not adept at using the app (e.g. how to submit LC symptoms experienced) may be unfairly underrepresented, as their data might be underrepresented or omitted altogether. Although a significant majority of individuals with LC reported symptoms multiple times (77%) which may suggest a high level of involvement and potential benefits from repeated usage, a notable portion (23%) reported symptoms only once, potentially suggesting a lack of sustained engagement with the app/this feature, or recovery from LC. Future studies into the reasons behind reporting symptoms only once could provide valuable insights for optimising engagement.

The exclusion of individuals without access to the app due to technological or socio-economic constraints (or lack of eligibility for other reasons) raises concerns about sample representativeness. This limitation could disproportionately affect certain demographic groups, such as older individuals, those with lower socio-economic status, individuals in areas with limited internet connectivity and/or individuals with severe symptoms that may not have been deemed clinically eligible for the DHI. Eligibility criteria for the LWCR DHI included proficiency in the English language, further excluding certain individuals with LC. We also acknowledge that some individuals using the LWCR DHI may not have felt able to accurately describe their symptoms and thus did not record symptoms or did not describe them as comprehensively as possible. We additionally acknowledge that this study concerns individuals with LC who were clinically expected to benefit from this intervention (and physically capable of rehabilitation), potentially excluding individuals with severe LC symptoms. Further work is required to gather insights from these populations.

## Conclusion

Our findings highlight the association between age, ethnicity and socio-economic factors and the severity of LC, calling for targeted interventions and support for vulnerable populations. Understanding these associations can inform healthcare policies and strategies aimed at minimising the burden of LC and improving patient outcomes.

Treatment for LC should focus on the most prevalent symptoms: pain, neuropsychological issues, fatigue and dyspnoea. Clinicians should be aware of and consider the wide range of other possible symptoms (e.g. palpitations or shivers) when developing treatment plans. It is crucial for LC clinics to maintain the capability to address the various symptomatic manifestations of LC. If these clinics face closure, they risk losing this essential ability, highlighting the need for sustained support to effectively manage LC's complexities.

**Figure 2.**
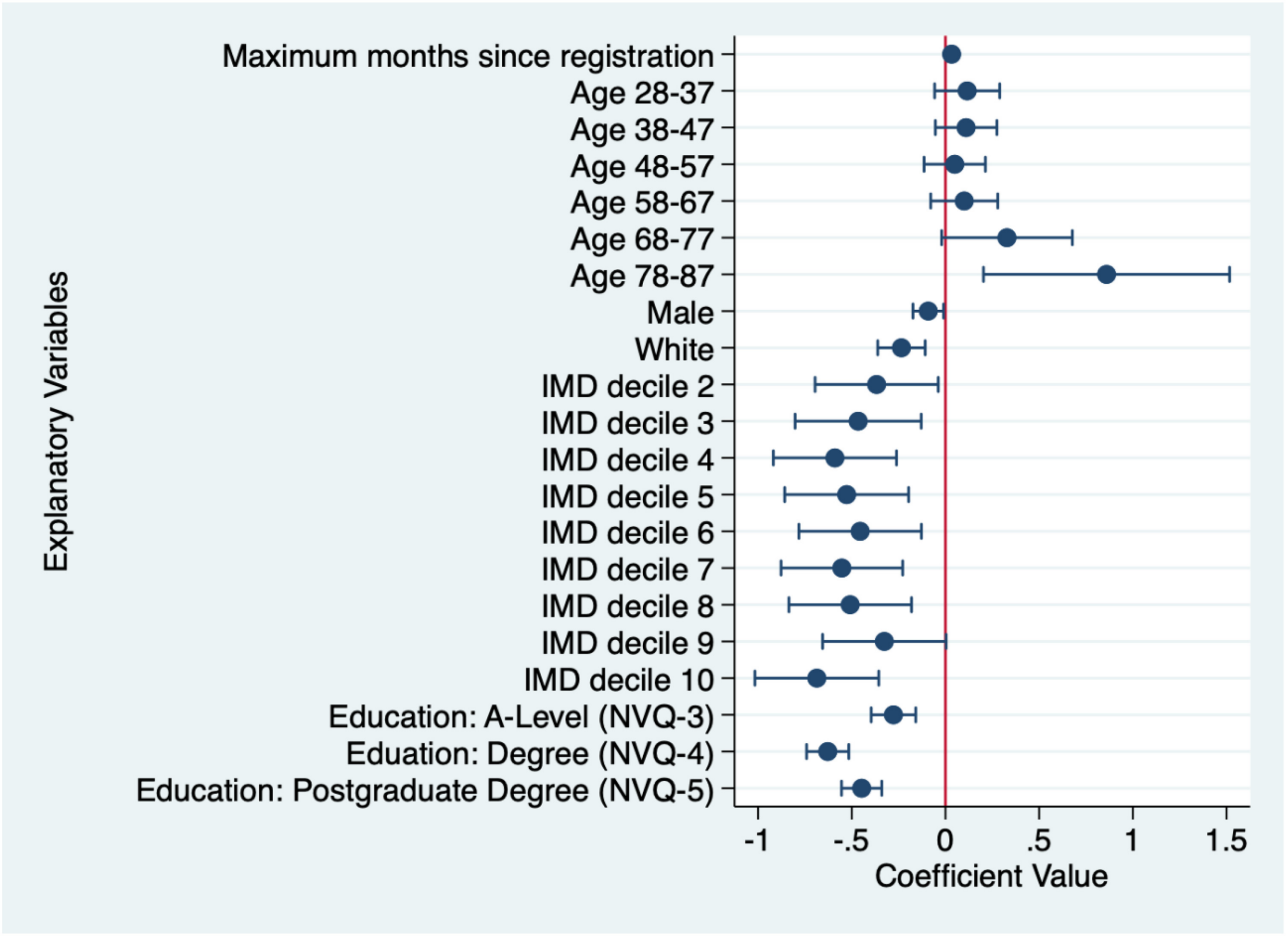
Regression results with 95% confidence interval.

*Note:* For age, the base group is people in the age category 18–27. For IMD, the base group is people from IMD decile 1. For education, the base group is people who left school before A-level (NVQ 1–2).

## Appendix

**Appendix 1.** Pain categories.

Abdominal pain
Arthralgia (joint pain)
Back pain
Chest pain
Dysmenorrhea (painful menstruation)
Eye pain
Eye pain/irritation
Facial dysaesthesia
Facial pain
Gingival pain
Headache
Jaw pain
Migraine
Myalgia (muscle pain)
Neck pain
Neuropathic pain
Neuropathic-type pain
Otalgia (ear pain)
Pain
Pleurisy (chest pain while breathing)

## Appendix

**Appendix 2.** Neuropsychological categories.

Anxiety
Anxiety / Breathless
Anxiety / Cognition
Anxiety / Depression
Anxiety / Fatigue
Anxiety / Fatigue / Breathless
Anxiety / Nerves
Anxiety / Pain
Clouding of Consciousness
Cognition
Depression
Depression / Anxiety
Derealisation
Disconnection
Dizziness/ Lightheaded
Embarrassment
Emotions
Frustration
Guilt
Nerves
Pain Nerves
Self-Harm
Sleep

## Appendix

**Appendix 3.** Symptom categories.

Symptom category	Count	Percentage of total symptoms reported (%)	Lowest intensity score reported	Highest intensity score reported
Pain	885	26.5	0	10
Neuropsychological	616	18.4	0	10
Fatigue	478	14.3	1	10
Dyspnoea	246	7.4	0	10
Palpitations	134	4	0	10
Light-headedness	94	2.8	0	10
Unclassifiable	75	2.2	0	10
Tinnitus	63	1.9	0	10
Paraesthesia	61	1.8	1	10
Insomnia	60	1.8	0	10
Nausea	57	1.7	2	10
Cough	54	1.6	0	10
Tremor	43	1.3	1	10
Diarrhoea	25	0.7	1	10
Sore throat	25	0.7	0	10
Vertigo	21	0.6	0	10
Rash	20	0.6	2	10
Dyspepsia	18	0.5	2	10
Visual disturbance	18	0.5	3	8
Pruritus	16	0.5	3	10
Pyrexia	16	0.5	2	10
Dysphonia	14	0.4	0	10
Dysgeusia	13	0.4	3	10
Anosmia	12	0.4	4	10
Dysosmia	11	0.3	1	10
Bloating	10	0.3	2	10
Joint stiffness	10	0.3	3	10
Limb weakness	10	0.3	0	9
Malaise	9	0.3	2	10
Pre-syncope/syncope	9	0.3	1	10
Coryzal	8	0.2	2	10
Hair loss	8	0.2	1	10
Ataxia	7	0.2	2	8
Night Sweat	7	0.2	2	10
Parosmia	7	0.2	3	10
Dysarthria	6	0.2	0	9
Feeling cold	6	0.2	7	10
Inflammatory	6	0.2	1	10
Myokymia	6	0.2	2	6
Amnesia	5	0.1	4	10
Aural Fullness	5	0.1	1	9
Dry eyes	5	0.1	2	9
Oral infection	5	0.1	4	10
Anorexia	4	0.1	5	9
Constipation	4	0.1	0	8
Dysphagia	4	0.1	7	9
Emesis	4	0.1	5	8
Enlarged lymph nodes	4	0.1	4	6
Flu-like symptoms	4	0.1	5	7
IBS flare	4	0.1	3	9
Immune	4	0.1	3	8
Myoclonus	4	0.1	3	10
Pedal oedema	4	0.1	3	10
Peripheral neuropathy	4	0.1	3	5
Photophobia	4	0.1	2	7
Swelling	4	0.1	4	9
Abdominal discomfort	3	0.1	4	10
Ageusia	3	0.1	7	10
Hypoxia	3	0.1	0	9
Loss of power in limbs	3	0.1	4	8
Muscular spasm	3	0.1	5	10
Myopathy	3	0.1	4	8
Rigors	3	0.1	5	7
Bradycardia	2	0.1	6	10
Crackles	2	0.1	5	8
Dizziness	2	0.1	5	7
Dysphasia	2	0.1	7	8
Haemoptysis	2	0.1	2	5
Hyperhidrosis	2	0.1	4	9
Infection	2	0.1	7	9
Nightmares	2	0.1	0	10
Photopsia	2	0.1	8	9
Poikilothermia	2	0.1	1	5
Problem with vision	2	0.1	5	7
Radiculopathy	2	0.1	7	7
Renal angle tenderness	2	0.1	6	8
Swollen glands	2	0.1	0	8
Symptoms similar to IBS flare	2	0.1	4	5
Urticaria	2	0.1	1	8
Walking difficulty	2	0.1	3	7
Abnormal sensation in limb	1	0	3	3
Akathisia	1	0	7	7
Cellulitis	1	0	4	4
Cramp	1	0	8	8
Cramping	1	0	5	5
Dysaesthesia	1	0	3	3
Facial paraesthesia	1	0	3	3
Fasciculations	1	0	9	9
Gastritis	1	0	6	6
Hearing loss	1	0	2	2
Hyperacusis	1	0	9	9
Hypertonia	1	0	2	2
Incontinence	1	0	3	3
Lhermitte's	1	0	5	5
Menorrhagia	1	0	10	10
Otitis Media	1	0	4	4
Poor concentration	1	0	10	10
Reflux	1	0	10	10
Rosacea	1	0	2	2
Seizure	1	0	10	10
Shivers	1	0	10	10
Sinusitis	1	0	1	1
Spasm	1	0	5	5
Sweating	1	0	8	8
Swollen lips	1	0	10	10
Symptoms of allergy	1	0	7	7
Symptoms similar to hypoglycaemia	1	0	9	9
Weight gain	1	0	10	10
Weight loss	1	0	1	1

## Appendix 4. Written Summary of Multiple Linear Regression Analysis.

The multiple linear regression model showed:

- The intensity of reported symptoms is positively associated with time between a patient's registration to the app and the initial reporting of symptoms (keeping all the other factors the same, that is, age, gender, ethnicity, IMD and education). When the time since registration and the initial reporting of symptoms increases by one month, the intensity of the symptom reported increases by 3.3% of s.d. (*p* < 0.01) (on a 0–10 scale, the intensity of the symptom reported increases by 0.1 (*p* < 0.01)).
- Age was significantly associated with intensity of symptoms (keeping other variables constant). Compared with age 18–27, age groups 68–77 and 78–87 were found to experience a higher intensity of symptoms. Specifically:
  - For people in age group 68–77, the intensity of symptoms experienced was 32.8% of s.d. (*p* < 0.1) higher (on a 0–10 scale, the intensity of symptoms was higher by 0.8 (*p* < 0.1)).
  - For people in age group 78–87, the intensity of symptoms experienced was 86% of s.d. (*p* < 0.05) higher (on a 0–10 scale, the intensity of symptoms was higher by 2.1 (*p* < 0.05)).
- Gender was significantly associated with intensity of symptoms (keeping other variables constant). For women, the intensity of symptoms experienced was 9.2% of s.d. (*p* < 0.05) higher than men (on a 0–10 scale, the intensity of symptoms was higher by 0.2 (*p* < 0.05)).
- Ethnicity was significantly associated with the intensity of symptoms (keeping other variables constant). For non-white individuals with LC, the intensity of symptoms experienced was 23.5% of s.d. (*p* < 0.01) higher than white individuals with LC (on a 0–10 scale, the intensity of symptoms was higher by 0.6 (*p* < 0.01)).
- Education was significantly associated with intensity of symptoms (keeping other variables constant). Compared with the least educated (NVQ 1–2), people with education level NVQ 3 to NVQ 5 experienced a lower intensity of symptoms. Specifically, compared with NVQ1–2:
  - For people with NVQ 3, the intensity of symptoms experienced was 27.7% of s.d. (*p* < 0.01) lower (on a 0–10 scale, the intensity of symptoms was less by 0.7 (*p* < 0.01)).
  - For people with NVQ 4, the intensity of symptoms experienced was 62.8% of s.d. (*p* < 0.01) lower (on a 0–10 scale, the intensity of symptoms was less by 1.5 (*p* < 0.01)).
  - For people with NVQ 5, the intensity of symptoms experienced was 44.7% of s.d. (*p* < 0.01) lower (on a 0–10 scale, the intensity of symptoms was less by 1.1 (*p* < 0.01)).
- IMD decile was significantly associated with intensity of symptoms (keeping other variables constant). Compared with the people who lived in the most deprived area (IMD = 1), all others were found to experience a lower intensity of symptoms. For example, compared with IMD decile 1:
  - For people in IMD decile 3, the intensity of symptoms experienced was 46.6% of s.d. (*p* < 0.01) lower (on a 0–10 scale, the intensity of symptoms was less by 1.1 (*p* < 0.01)).
  - For people in IMD decile 5, the intensity of symptoms experienced was 52.7% (*p* < 0.01) lower (on a 0–10 scale, the intensity of symptoms was less by 1.3 (*p* < 0.01)).
  - For people in IMD decile 10, the intensity of symptoms experienced was 68.6% of s.d. (*p* < 0.01) lower (on a 0–10 scale, the intensity of symptoms was less by 1.7 (*p* < 0.01)). All IMD deciles are displayed graphically in Figure 2.
- No statistically significant association was found between IMD decile and number of symptoms.
